# Long-term follow-up results of topical imiquimod for high-grade cervical intraepithelial neoplasia in young women: an original research

**DOI:** 10.1186/s12905-026-04685-8

**Published:** 2026-08-14

**Authors:** Jungmi Kim, Ju-Hyun Kim, Yaesol Kim, Rumie Lee, Hyeonji Choi, Dae-Yeon Kim

**Affiliations:** 1 Department of Obstetrics and Gynecology, Chungbuk National University Hospital, Chungbuk National University College of Medicine, Cheongju, Republic of Korea; 2 Department of Obstetrics and Gynecology, Asan Medical Center, University of Ulsan College of Medicine, 88, Olympic-ro 43-gil, Songpa-gu, Seoul, 05505 Republic of Korea; 3 Department of Obstetrics and Gynecology, Chungbuk National University Hospital, Cheongju, Republic of Korea

**Keywords:** Imiquimod, Uterine Cervical Dysplasia, Human Papillomavirus, Retrospective Studies, Demography, Fertility

## Abstract

**Background:**

Cervical excisional treatment is associated with adverse obstetric outcomes. Although imiquimod has been explored as a conservative alternative in trials like TOPIC-3, its definitive role remains debated. We aimed to evaluate the long-term efficacy of a clinician-applied imiquimod strategy specifically in women aged under 35 years to verify its potential for fertility preservation.

**Methods:**

We conducted a retrospective, single-arm cohort study of nulliparous women aged under 35 years with Cervical Intraepithelial Neoplasia (CIN) 2 or 3 and no prior excisional therapy between October 2021 and March 2024. The primary outcomes were histological regression assessed immediately post-treatment (8 weeks) and during a prolonged follow-up period.

**Results:**

A total of 100 patients were included. The immediate regression rate following the 8-week imiquimod course was 96.0%, with an initial high risk Human Papillomavirus (hr-HPV) clearance rate of 57.1%. In the follow-up analysis, the sustained regression rate reached 91.0%, and the cumulative hr-HPV clearance rate rose to 70.5%. Notably, patients with CIN2 without HPV 16/18 achieved the highest regression rate (96.5%), successfully avoided or postponed surgical intervention.

**Conclusions:**

The clinician-applied imiquimod strategy offers a robust therapeutic impact, safely postponing surgical intervention for approximately one year in reproductive-aged women with CIN 2/3. This imiquimod strategy serves as an effective bridging therapy that preserves fertility without compromising clinical safety, providing a supportive clinical decision-making model for individualized non-surgical management.

## Background

 Recent demographic data indicate a declining trend in the overall incidence of cervical cancer in South Korea. However, a disproportional burden remains among young women. The prevalence among women aged 15 to 34 remains 4.4 per 100,000 persons, ranking cervical cancer as one of the most common malignancies in this age group. This sustained burden of disease in young women is often attributed to shifting behavior patterns, such as an earlier age of coitarche, which increases the duration of exposure to human papillomavirus(HPV) [1]. As the maternal age at first pregnancy continues to rise, the incidence of high-grade cervical intraepithelial neoplasia (CIN) encountered prior to or during the first pregnancy has also increased. According to the 2019 American Society for Colposcopy and Cervical Pathology guidelines, observation is recommended for women under 25 years of age diagnosed with CIN2. For the patients diagnosed with CIN3, diagnostic excisional therapy is recommended regardless of their age [2]. 

However, observations alone are not always a sufficient strategy. A recent long-term study involving women aged 21–39 diagnosed with CIN2 showed that 30% eventually required treatment due to persistent disease [3]. Furthermore, excisional therapy carries an inherent risk of preterm birth, although its influence is controversial. A 2017 Cochrane review showed that electrosurgical treatment significantly increases the relative risks of preterm birth (< 37 weeks), preterm premature rupture of membranes, and low birth weight (< 2.5 kg) with relative risks of 1.75, 2.36, and 1.81, respectively [4–8]. Thus, therapeutic methods that are more active than simple observation yet less invasive than excisional therapy are urgently required for women of child-bearing age [9]. 

Topical imiquimod, an immune response modifier and toll-like receptor 7 and 8 (TLR7/8) agonist, has been investigated as a potential cervix-preserving alternative. By activating TLR7 and the subsequent NF-κB signaling pathway, imiquimod bypasses the inhibitory effects of HPV-derived E6 and E7 oncoproteins, promoting the production of pro-inflammatory cytokines such as interferon-alpha (IFN-α) and tumor necrosis factor-alpha (TNF-α) while enhancing cellular immunity [10]. 

Despite its pharmacological potential, previous clinical trials have reported that imiquimod may be less effective than electrosurgical procedures [11–14]. For instance, the TOPIC-3 study showed a success rate of 60% for imiquimod compared to 95% for large loop excision of the transformation zone (LLETZ) [15]. Similarly, a Phase 3 trial reported a histological regression rate of 63% at 6-month post-treatment, and a 2023 meta-analysis found regression rates of 55% of imiquimod versus 93% for surgery. [16–17] Therefore, we aimed to evaluate the long-term efficacy of a clinician-applied imiquimod strategy in a focused cohort of women aged < 35 years, identifying the optimal clinical window where imiquimod can serve as a viable alternative to excisional treatment.

## Methods

The study was designed as a retrospective cohort study and was conducted at a single center in the Republic of Korea, between October 2021 and March 2024. The protocol of this study was reviewed and approved by the Institutional Review Board of this institution (approval number: 2025 − 1093, approval date: 1st September 2025). Informed consent was waived because the study was retrospective in design. We obtained data of patients who were nulliparous, between the ages of 20 and 35 years, had never received cervical excisional therapies, and were concerned about future pregnancy outcomes. All patients were carefully confirmed to have CIN 2 or 3 through multiple cervical punch biopsies performed by gynecologic oncologists. Patients who did not complete the 8-week treatment due to these adverse effects were excluded from the analysis. All patients were fully informed that topical imiquimod application was not the standard treatment for high-grade squamous intraepithelial lesions (HSIL) at this age and had time to consider their decision.

**Fig. 1:**
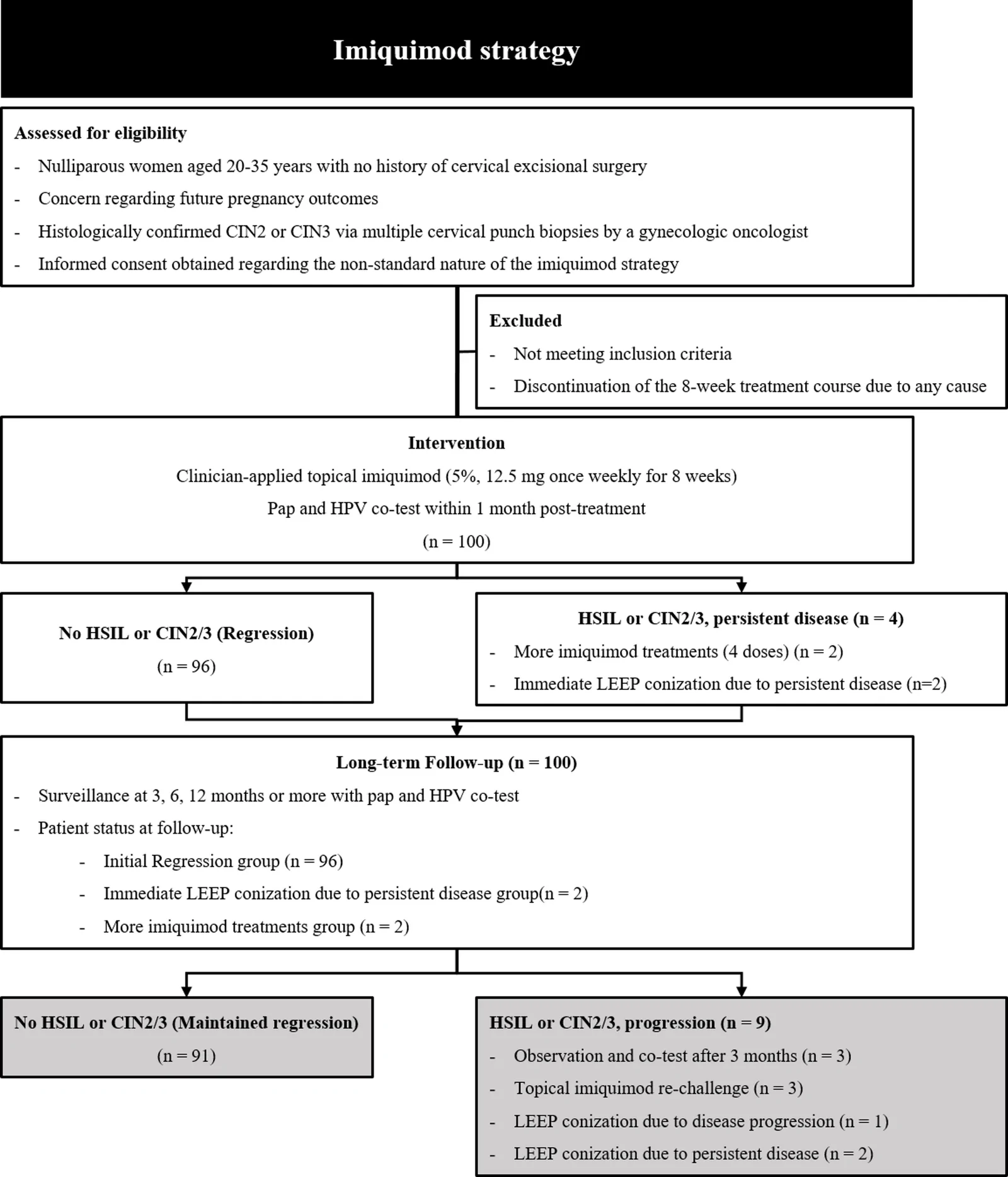
The brief summary of treatment course we use in this journal named “imiquimod strategy”. Abbreviations: LEEP, Loop Electrosurgical Excision procedure; CIN, Cervical intraepithelial Neoplasia; HPV, Human Papillomavirus; HSIL, High-Grade Squamous Intraepithelial Lesion

In our “imiquimod strategy,” all participants received imiquimod treatment once weekly for eight weeks. A 12.5 mg dose of 5% imiquimod cream was applied by gynecologic oncologists using a brush-type cervical applicator. The eight-week treatment duration was determined based on patient compliance and side effects reported in a previous study by Juhyun Kim et al. [18] To minimize vaginal irritation, we recommended that patients use a vaginal menstrual cup after administration and remove it the following morning. The occurrence and severity of side effects were assessed at each visit. Adequate contraception was ensured throughout the study period. Pap and HPV co-testing with colposcopy (including biopsies) were performed at 1, 3, 6, and 12 months following the last week of treatment. Punch biopsies or endocervical curettages were performed when suspicions of CIN2/3 lesions were observed. If the immediate results showed persistent HSIL or CIN 2/3, the patient was consulted to determine the next step, which included either immediate excisional therapy or additional imiquimod treatment. (Fig. 1).

Gynecologic pathologists conducted histopathological assessments of cervical biopsies in accordance with national guidelines and World Health Organization criteria, utilizing hematoxylin and eosin staining, as well as p16 and Ki-67 immunostaining. High-risk HPV cases were subtyped for genotypes 16, 18, 31, 33, 35, 39, 45, 51, 52, 56, 58, 59, 66, and 68 using real-time polymerase chain reaction/tagging oligonucleotide cleavage and extension, multiple detection temperatures, and three cycle threshold methods, employing the Allplex HPV28 Detection Kit (Seegene Inc., Seoul, Korea). Low-risk HPV cases were subtyped for genotypes 6, 11, 26, 40, 42, 43, 44, 53, 54, 61, 69, 70, 73, or 82 using the same methodology.

The primary endpoint was the regression rate after 8-week topical imiquimod, and the secondary endpoints included hr-HPV clearance rate, progression rate, and long-term pregnancy outcomes. “Complete remission” was defined as (1) no identifiable CIN lesions or any suspicious lesions on colposcopy conducted by a single dedicated gynecologic oncologist, and (2) a concurrent negative cytology result. “Regression” was defined as any clinical down-staging o from baseline, including all cases of complete remission, histological CIN 1, or absence of CIN-suspicious lesion on colposcopy with concurrent Low-grade Squamous intra-epithelial Lesion(LSIL) or Atypical Squamous Cells of Undetermined Significance(ASC-US) cytology. “Persistent disease” was defined as exhibiting atypical squamous cells—cannot exclude high-grade squamous intraepithelial lesion (ASC-H)—or HSIL on cytology within a month post-treatment, with CIN2/3 confirmed by additional colposcopic biopsy at that time. Due to the short interval from initial diagnosis, these cases were classified as persistence rather than progression, representing true therapeutic non-responsiveness. This time-point served as an immediate decision gate to determine whether to extend the topical imiquimod therapy or crossover to the definitive loop electrosurgical excision procedure (LEEP). In contrast, “Progression” was defined as the identification of ASC-H or HSIL on cytology, with CIN2/3 confirmed by colposcopic biopsy, during the 3-, 6-, or 12-month follow-up window, in patients whose initial within a month post-treatment evaluation showed no evidence of CIN2/3. These cases represented patients who initially responded to imiquimod therapy. At this stage, subsequent management strategies-including active observation, an additional 4 or 8 doses of imiquimod, or LEEP conization-were determined through shared decision-making with the patients. No patients in progression groups advanced to invasive cervical cancer during the study period. “High-risk HPV clearance” was defined as the absence of high-risk HPV detection or the absence of high-risk HPV detection (i.e., only low-risk HPV detected) on co-testing performed with high-risk HPV infection confirmed at the initial examination. Subgroup analysis was performed to determine the differences in treatment outcomes based on the presence or absence of specific HPV types (HPV-16 and HPV-18) and initial histological results using appropriate statistical tools.

### Statistical analysis

After patient recruitment, the relevant participants were counted to determine the primary and secondary outcomes. To ensure the robustness and transparency of the data, all 100 enrolled patients were included in the long-term outcome analysis according to the Intention-to-Treat (ITT) principle. Fisher’s exact test was used to analyze differences in regression rates by the presence of HPV 16 or HPV 18 and initial histologic grade. It was also used to evaluate the association between early hr-HPV clearance and subsequent clinical regression. Progression-free survival (PFS) and cumulative hr-HPV clearance in long-term follow-up data were analyzed using the Kaplan-Meier method. For the analysis of hr-HPV clearance, the nine patients with missing baseline data were excluded from the denominator. All statistical analyses were conducted using the IBM SPSS Software for Windows, version 25 (IBM Corp., Armonk, NY, USA).

## Results

One hundred patients were enrolled in the study between October 2021 and March 2024. Seventy-four (74.0%) patients had CIN2, while the remaining 26 (26.0%) had CIN3. Additionally, 27 (27.0%) patients tested positive for HPV types 16 or 18, whereas 64 (64.0%) patients had other hr-HPV types. Among the 100 enrolled patients, nine were excluded from the high-risk HPV clearance analysis due to missing baseline HPV data. Although these patients had a documented history of hr-HPV infection from external clinics prior to imiquimod treatment, they were excluded to maintain analysis consistency because their baseline HPV status was unavailable within our institution’s records or through a repeated test. Consequently, clinical regression was analyzed for the total cohort(*n* = 100), while the HPV clearance rate was calculated based on 91 patients with confirmed baseline positivity. The baseline characteristics are presented in Table 1.

**Table 1:** Patient characteristics

Variables	No. of Patients (*N* = 100)
Age(years)	
20–24	17
25–35	83
High risk HPV infection	91
with HPV 16 or 18 infection	27
with other high risk HPV infection	64
Initial punch biopsy result	
CIN2	74
CIN3	26

*Abbreviations*: *HPV* Human papillomavirus, *CIN* Cervical intraepithelial neoplasia

### The immediate outcome was observed one month after treatments

The initial results observed one month after completing eight weeks of imiquimod treatment are presented in Table 2. Eighty patients (80.0%) achieved complete remission to normal, while 96 participants (96.0%) regressed to CIN1 or lower. The regression rate was 88.9% (24/27) in patients with hr-HPV 16 or 18, whereas it was 98.6% (72/73) in those without these genotypes. Although this difference did not reach statistical significance (*P*=0.059, Fisher exact test), a numerical trend toward a lower regression rate was observed in the HPV 16/18-positive group (OR = 0.111; 95% CI, 0.011–1.119). Histologic grade (CIN2 versus CIN 3) showed no association with the likelihood of regression during the study period. (OR = 0.947; *P* > 0.999).

**Table 2:** The immediate outcome observed within one month after eight weeks of weekly topical imiquimod applications

Results	No. of patients (*N* = 100)	*p*-value
Complete remission	80 (80.0%)	
Regression(≤ LSIL)	96 (96.0%)	
Regression with HPV 16 or 18	24 (24/27, 88.9%)	*P* = 0.059
Regression without HPV 16 or 18	72 (72/73, 98.6%)	
Regression from CIN2	71 (71/74, 95.9%)	*P* = 1.000
Regression from CIN3	25 (25/26, 96.2%)	
hr-HPV clearance	52 (52/91*, 57.1%)	
with HPV 16 or 18	16 (16/27, 59.3%)	*P* = 0.821
with other high risk HPV infection	36 (36/64, 56.3%)	
Persistent disease	4 (4.0%)	
who wants more imiquimod treatment	2	
who wants LEEP	2^†^	

Complete Remission: No CIN on colposcopy and negative cytology; Regression: CIN1 or lower on colposcopy with LSIL, ASC-US, or negative cytology; Persistent disease: persistent CIN2/3 on colposcopy with ASC-H or HSIL cytology; hr-HPV clearance, absence of hr-HPV on co-testing (includes cases with only low-risk HPV detection)

In the table, the figures in % in parentheses are the percentage of the total patient population

*Abbreviations*: *LSIL* Low-grade squamous intraepithelial lesion, *HPV* Human papilloma virus, *CIN* Cervical intraepithelial neoplasia, *LEEP* Loop Electrosurgical Excision procedure, *ASC-US*, Atypical squamous cells of undetermined significance, *ASC-H* Atypical squamous cells- cannot exclude high-grade squamous intraepithelial lesion, *HSIL* High-grade squamous intraepithelial lesion, *hr-HPV* High-risk human papillomavirus

†The biopsy results of excisional therapy; one was normal and the other was CIN3

*Nine patients with missing baseline status were excluded

Immediate hr-HPV clearance was observed in 52 of 91 patients (57.1%). The clearance rate was 59.3% for HPV 16/18 and 56.3% for other hr-HPV(*P*=0.821). Fisher’s exact test revealed no significant difference in the HPV clearance rate between the HPV 16/18-positive and negative groups (odds ratio 1.131; *p* = 0.821). In particular, only four patients (4.0%) exhibited persistent HSIL. Among them, two patients received an additional four imiquimod treatments, while the other two underwent immediate LEEP conization; pathological findings from these excisions confirmed normal tissue in one case and CIN3 in the other.

### Long-term follow-up results

**Fig. 2:**
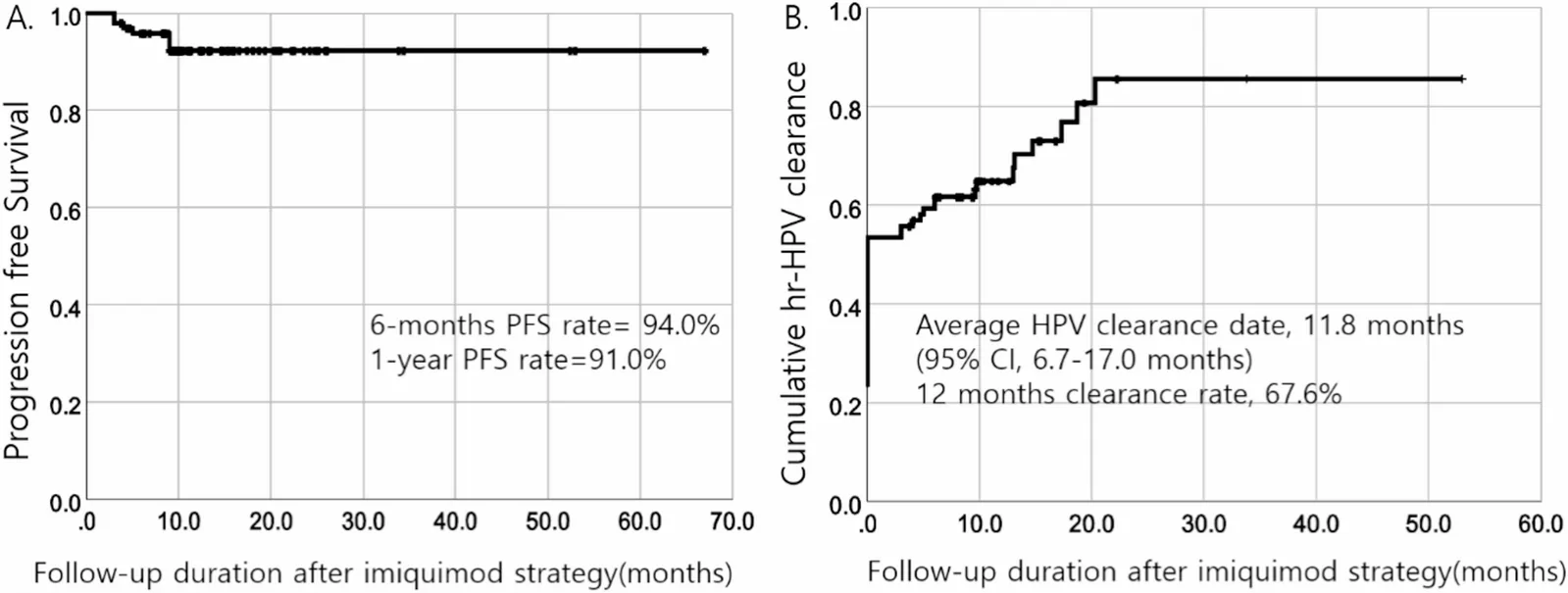
Progression-free survival and cumulative hr-HPV clearance during follow-up after implementing the imiquimod strategy. **A** Progression free survival curve after imiquimod strategy. **B** Cumulative hr-HPV clearance after imiquimod strategy. Abbreviations: HPV, Human Papillomavirus

The average follow-up duration was 438 days, with a median of 327 days (range: 110–2011 days). Following the ITT principle, 80.0% of the total cohort achieved complete remission, and 91.0% showed regression to a level lower than CIN 1. (Table 3.). Among the 91 patients who achieved regression, one patient reached this state after receiving four additional imiquimod applications following an initial persistent HSIL finding. While the presence of HPV 16/18 showed a potential trend toward lower regression (*P*=0.057; OR = 0.255), early hr-HPV clearance immediately after the 8-week treatment emerged as a strong positive indicator of subsequent clinical regression. Among patients with HPV 16 or 18, those who achieved early clearance were four times more likely to reach regression compared to those with persistent hr-HPV (OR = 4.0; 95% CI, 0.584–27.411; *P*=0.187). Specifically, regression was observed in 87.5%(14/16) of patients with early clearance versus 63.6% (7/11) with persistent viral status. Kaplan-Meier analysis demonstrated robust maintenance of progression-free status, with a 1-year progression-free rate of 0.91. The cumulative hr-HPV clearance rate increased to 70.5% during follow-up, with an estimated average duration for clearance of 11.8 months (95% CI, 6.7–17.0 months). This information is illustrated in Fig. 2.

**Table 3:** Long-term follow-up results of imiquimod strategy

Results	No. of patients (*N* = 100)	*p*-value
Complete remission	80 (80.0%)	
Regression	91 (91.0%)	
Regression with HPV 16 or 18	22 (22/27, 88.0%)	*P* = 0.057
Regression without HPV 16 or 18^a^	69 (69/73, 94.5%)	
Regression from CIN2	69 (69/74, 93.2%)	*P* = 0.233
Regression from CIN3	22 (22/26, 84.6%)	
hr-HPV clearance by imiquimod strategy	62 (62/88^b^, 70.5%)	
with HPV 16 or 18	17 (17/25, 68.0%)	*P* = 0.798
with other high risk HPV infection	45 (45/63, 71.4%)	
Number of persistent or progression	9 (9.0%)	
who wants observation	3^c^	
who wants more imiquimod treatment	3^d^	
who wants LEEP	3^**e**^	
Pregnancies achieved post-treatment		
Full-term	4 (4.0%)	
Pre-term	0	

Complete Remission: No CIN on colposcopy and negative cytology; Regression: CIN1 or lower on colposcopy with LSIL, ASC-US, or negative cytology; Progression: confirmation of CIN2/3 via colposcopic biopsy with concurrent ASC-H or HSIL cytology; hr-HPV clearance, absence of hr-HPV on co-testing (includes cases with only low-risk HPV detection)

In the table, the figures in % in parentheses are the percentage of the total patient population

*Abbreviations*: *HPV* Human papillomavirus, *CIN* Cervical intraepithelial neoplasia, *hr-HPV* High-risk human papillomavirus, *LEEP* Loop electrosurgical excision procedure, *LSIL* Low-grade squamous intraepithelial lesion, *ASC-US *Atypical squamous cells of undetermined Significance, *ASC-H* Atypical squamous Cells- cannot exclude high-grade squamous intraepithelial lesion, *HSIL* High-Grade squamous intraepithelial lesion

^a^“Regression without HPV 16/18” group is a heterogeneous cohort encompassing patients with non-16/18 high-risk HPV types or missing initial HPV status

^b^Excluding 9 patients with missing baseline HPV status and 3 who received LEEP

^c^All of them were regressed without any further treatments

^d^Two of them got four more imiquimod treatments, and the other got eight more imiquimod treatments

^e^Two with persistent disease immediately post-treatment; 1 with progression during follow-up (confirmed CIN3)

During the follow-up, nine patients (9.0%) exhibited persistent or progressive disease.

Following the method, ‘progression’ was strictly defined as the histological confirmation of CIN 2/3 after regression; specifically, no cases of progression to invasive cervical cancer were observed throughout the study period. Of these, three achieved regressions through observation alone, and another three regressed after supplemental imiquimod applications. The remaining three (3.0%) eventually required excisional therapy; specifically, two of these surgical cases were those who had undergone immediate LEEP due to persistent disease. (Fig. 1.) Subgroup analysis revealed that the highest regression rate (96.5%) was achieved in patients with CIN2 without HPV type 16 or 18. (Table 4.) In a focused analysis of 44 patients followed for more than 360 days, the regression rate remained high at 90.9% (40/44), with only two patients requiring excisional therapy during this extended period.

**Table 4:** Regression rate based on HPV and initial histologic type in long-term follow-up period

	With HPV 16 or 18	Without HPV 16 or 18*
CIN2	82.4% (14/17)	96.5% (55/57)
CIN3	80.0% (8/10)	87.5% (14/16)

*Abbreviations*: *HPV* Human papillomavirus, *CIN* Cervical intraepithelial neoplasia

*“Regression without HPV 16/18” group is a heterogeneous cohort encompassing patients with non-16/18 high-risk HPV types or missing initial HPV status

## Discussion

This study demonstrates that a clinician-applied imiquimod strategy is highly beneficial for patients under 35 years of age with CIN 2/3 who have not undergone prior cervical excisional surgery, particularly regarding the preservation of fertility. In previous trials such as TOPIC-3 trial by Hendriks et al. and the concurrent study by Polterauer et al., self-applied imiquimod cream (3 times a week for 16 weeks) resulted in a regression rate of approximately 60% [15, 16]. While most existing studies have often utilized self-applied vaginal imiquimod for 8 to 16 weeks, such suppositories are currently unavailable in South Korea. Furthermore, a three-times a week clinical visitation schedule is practically unfeasible for most patients [15, 16, 19, 20]. Remarkably, our imiquimod strategy-utilizing clinician-applied imiquimod only once weekly only 8 weeks-resulted in a regression rate of 91%. In a recent systematic review by Galio et al. highlighted that no published studies had focused on the role of imiquimod in HIV-negative patients. And in the same journal, a lower complete response rate has been reported for intra-anal HSIL compared with peri-anal HSIL, suggesting that the efficacy of topical imiquimod is highly dependent on the accessibility of the lesion [21, 22]. The superior efficacy observed in our cohort suggests that direct clinician application may ensure better drug delivery to internal cervical lesions.

The regression and hr-HPV clearance rates observed in our study exceed those typically reported for observation in previous literature. For instance, Grimm et al. reported a 39% histologic regression rate and 14% hr-HPV clearance rate in their placebo group, while Sheth et al. observed 79% and 50%, respectively [20, 23–25]. A notable finding in our cohort was progressive increase in the hr-HPV clearance rate, which rose from 57.1% immediately post-treatment to 70.5% during long-term follow-up, suggesting a lasting immunologic effect on the cervical microenvironment. The sustained impact of imiquimod on viral clearance was further supported by Kaplan-Meier analysis, which demonstrated a cumulative increase in hr-HPV clearance over time. (Fig. 2.) These findings suggest that imiquimod treatment may offer a therapeutic advantage over simple observation by actively promoting both higher regression rates and more robust viral clearance.

Our analysis revealed a consistent trend toward lower regression rates in the presence of HPV 16 or 18, with aligned odds ratios observed in both Table 2 (OR = 0.111; 95% CI, 0.011–1.119; *P*=0.059) and Table 3(OR = 0.255; 95% CI, 0.063–1.034; *P*=0.057). While these findings consistent with previous reports suggesting higher resistance in these genotypes [18], it is noteworthy that a high regression rate of 88.9% was still achieved within this high-risk subgroup. Furthermore, achieving early hr-HPV clearance immediately following the 8-week imiquimod treatment emerged as strong positive predictor of clinical regression. In our cohort, patients who achieved early clearance were four times more likely to reach successful regression compared to those with persistent hr-HPV (OR = 4.0; *P*=0.187). Although this trend did not reach statistical significance, it underscores the importance of post-treatment viral status as a primary indicator for long-term therapeutic success. Our findings confirm that even in the presence of HPV 16 or 18, the clinician-applied imiquimod strategy remains a viable option.

It is noteworthy that clinical regression can still be achieved with a suboptimal initial response. In our cohort, one patient who exhibited persistent HSIL immediately after the 8-week imiquimod course reached regression after receiving four additional applications. Similarly, three patients who showed disease progression were managed with a re-challenge of imiquimod (4 or 8 additional applications), all of whom subsequently maintained regression. Unlike surgical excision, which is difficult to repeat due to cervical tissue loss and obstetric risks, imiquimod can be re-applied to trigger a renewed immune response. This highlights its potential as a repeatable, conservative intervention to regain disease control. While these observations are based on a limited number of cases, they suggest that in carefully selected young patients, a second course of imiquimod can be considered an effective conservative approach to regain disease control and further delay or avoid invasive surgery.

Our 1-year follow-up data demonstrated an overall regression rate of 91%, which compares favorably with previously reported rates [15, 19]. During this period, only 3% of the total cohort eventually required cervical excisional therapy, and no one progressed to invasive cancer. Importantly, 4% of the patients achieved full-term delivery following the “imiquimod strategy”. Kaplan-Meier analysis further supported these long-term outcomes, showing a 6-month PFS rate of 94.0% and a 1-year PFS rate of 91.0%. Furthermore, among the 44 patients followed for more than 360 days, 40 achieved regression (90.9%), suggesting that the therapeutic efficacy of this clinician-applied strategy is not only robust but also well-sustained post-treatment. These findings gain further significance when considering the long-term recurrence risks associated with surgical intervention. Serati et al. reported a median time to recurrence after conization of 12.4 months, with 13.8% of patients requiring a second excisional procedure or even hysterectomy [26]. In this context, delaying the initial cervical resection by approximately one year through our imiquimod strategy could serve as an effective bridging therapy.

Several limitations should be considered when interpreting our results. First, the lack of a concurrent control group-either a spontaneous regression cohort or a group undergoing immediate cervical excisional treatment-makes it challenging to isolate the independent therapeutic effect of imiquimod. Second, our findings may be subject to self-selection bias; the study cohort likely consisted of highly motivated individuals who complete the full 8-week regimen, whereas those with perceived higher risks, such as HPV 16/18 or CIN3, might have opted for surgical intervention. Third, the high-risk HPV clearance analysis included a small subset of patients with missing baseline hr-HPV data, which may have introduced heterogeneity into the results. Finally, the relatively small sample size precluded a detailed subgroup analysis by age, such as comparing patients under 25 with those aged 25 to 35. Consequently, these results may not be fully representative of the entire population of young women with high-grade CIN. Despite these limitations, our study provides meaningful real-world evidence regarding the clinical utility of a clinician-applied imiquimod strategy in a conservative management setting.

Therefore, a randomized prospective study comparing spontaneous regression, the “imiquimod strategy”, and cervical excisional treatment is warranted to further validate the efficacy of imiquimod in delaying invasive procedures. Future research with a larger cohort and an extended follow-up period could more definitively evaluate live birth rates and provide granular comparisons between age subgroups, such as patients under 25 versus those aged 25 to 35.

## Conclusion

In conclusion, this study is the first to provide long-term(1-year) follow-up data on clinician-applied imiquimod strategy for young women with high-grade CIN. By achieving a sustained regression rate of 90% alongside a 70.5% high-risk HPV clearance rate, and limiting the necessity for excisional surgery to only 3% of the total cohort (3/100), we demonstrate that imiquimod can serve as a robust bridging therapy. Our finding confirms that for selected young patients who prioritize future fertility, the imiquimod strategy offers a safe and effective clinical decision-making model that successfully postpone cervical excisional procedures without compromising oncologic safety. This research provides a pioneering foundation for shifting toward more individualized, non-surgical management of CIN2/3 in reproductive-age women. 

## Data Availability

The datasets analyzed during this study are available from the corresponding author upon reasonable request.

## Abbreviations

CIN: Cervical intraepithelial neoplasia
HPV: Human papillomavirus
hr: HPV–high risk human papillomavirus
ASC: US–atypical squamous cells of undetermined significance
LSIL: Low–grade squamous intraepithelial lesion
ASC: H–Atypical squamous cells–cannot exclude high–grade squamous intraepithelial lesion
HSIL: High–grade squamous intraepithelial lesion
LEEP: Loop electrosurgical excision procedure
LLETZ: Large loop excision of the transformation zone
